# Extracorporeal Life Support in Myocardial Infarction: New Highlights

**DOI:** 10.3390/medicina60060907

**Published:** 2024-05-30

**Authors:** Giulia Piccone, Lorenzo Schiavoni, Alessia Mattei, Maria Benedetto

**Affiliations:** 1 Cardiothoracic and Vascular Intensive Care Unit, Hospital and University Trust of Verona, P. le A. Stefani, 37124 Verona, Italy; giulia.piccone@gmail.com; 2 Department of Anesthesia and Intensive Care, Fondazione Policlinico Universitario Campus-Bio-Medico, Via Alvaro del Portillo 200, 00127 Roma, Italy; a.mattei@policlinicocampus.it; 3 Cardio-thoracic and Vascular Anesthesia and Intensive Care Unit, IRCCS Azienda Ospedaliero-Universitaria di Bologna, Via Albertoni 15, 40138 Bologna, Italy; mariabenedettoanestesista@gmail.com

**Keywords:** cardiogenic shock, veno-arterial extracorporeal membrane oxygenation, acute myocardial infarction, mechanical circulatory support, left ventricular unloading, Impella, weaning, intra-aortic balloon pump

## Abstract

*Background and Objectives*: Cardiogenic shock (CS) is a potentially severe complication following acute myocardial infarction (AMI). The use of veno-arterial extracorporeal membrane oxygenation (VA-ECMO) in these patients has risen significantly over the past two decades, especially when conventional treatments fail. Our aim is to provide an overview of the role of VA-ECMO in CS complicating AMI, with the most recent literature highlights. *Materials and Methods*: We have reviewed the current VA-ECMO practices with a particular focus on CS complicating AMI. The largest studies reporting the most significant results, i.e., overall clinical outcomes and management of the weaning process, were identified in the PubMed database from 2019 to 2024. *Results:* The literature about the use of VA-ECMO in CS complicating AMI primarily has consisted of observational studies until 2019, generating the need for randomized controlled trials. The EURO-SHOCK trial showed a lower 30-day all-cause mortality rate in patients receiving VA-ECMO compared to those receiving standard therapy. The ECMO-CS trial compared immediate VA-ECMO implementation with early conservative therapy, with a similar mortality rate between the two groups. The ECLS-SHOCK trial, the largest randomized controlled trial in this field, found no significant difference in mortality at 30 days between the ECMO group and the control group. Recent studies suggest the potential benefits of combining left ventricular unloading devices with VA-ECMO, but they also highlight the increased complication rate, such as bleeding and vascular issues. The routine use of VA-ECMO in AMI complicated by CS cannot be universally supported due to limited evidence and associated risks. Ongoing trials like the Danger Shock, Anchor, and Recover IV trials aim to provide further insights into the management of AMI complicated by CS. *Conclusions*: Standardizing the timing and indications for initiating mechanical circulatory support (MCS) is crucial and should guide future trials. Multidisciplinary approaches tailored to individual patient needs are essential to minimize complications from unnecessary MCS device initiation.

## 1. Background

Cardiogenic shock (CS) is a potential life-threatening complication occurring after acute myocardial infarction (AMI), with an overall incidence of 60%. Although it has been demonstrated that early primary revascularization improves 6-month clinical outcomes, mortality due to CS occurring after AMI still ranges from 40% to 50% [1,2].

This happens because of a sudden and profound decrease in the heart’s ability to provide adequate oxygen delivery to vital organs. The direct consequence is multiorgan failure.

Several hemodynamic factors may contribute to this condition, including (1) decreased cardiac output; (2) increased systemic vascular resistances (SVR) as a compensatory mechanism to the decreased cardiac output (CO), which can lead to an increased myocardial afterload; (3) reduced stroke volume (SV) due to decreased myocardial contractility or impaired ventricular filling; (4) increased pulmonary capillary wedge pressure (PCWP), which may lead to pulmonary congestion; and (5) impaired tissue perfusion which can lead to end organ dysfunction, if not promptly addressed.

Managing CS typically consists of identifying the underlying cause, optimizing cardiac preload and afterload, and supporting the cardiac function with inotropes and or vasoactive drugs. If CS becomes refractory to conventional therapy, establishing a short-term mechanical circulatory support (MCS) should be considered [3].

Short-term MCS support includes an intra-aortic balloon pump (IABP), ventricular unloading systems such as Impella, and veno-arterial extracorporeal membrane oxygenation (VA-ECMO).

The intra-aortic balloon pump in cardiogenic shock II (IABP-Shock II) trial, involving 600 patients with AMI complicated by CS, found that the use of the IABP did not lead to a significant decrease in 30-day all-cause mortality (39.7% versus 41.3%; *p* = 0.69). As a result of these findings, the routine use of IABP is not recommended [4].

Despite the lack of evidence, there has been a significant increase in the use of VA-ECMO for patients with CS (from 0.1% to 3%) over the last two decades.

VA-ECMO has become the preferred MCS in patients with CS, and it has been widely used in several settings of CS (AMI, acute or chronic heart failure, myocarditis, septic cardiomyopathy, graft failure after heart transplantation, chronic and acute right ventricular failure, pulmonary embolism, postcardiotomy syndrome). More specifically, VA-ECMO has emerged as a crucial and game-changing intervention in managing CS occurring after myocardial infarction [5,6].

It plays several roles in this critical scenario, including (1) rapid hemodynamic support by temporarily substituting the heart’s function and providing adequately oxygenated blood to vital organs; (2) a bridge to recovery, allowing the heart to rest and heal, especially in cases in which the myocardium is damaged and conventional treatments may not be sufficient; (3) a bridge to decision; and (4) a bridge to heart transplant or durable MCS implantation.

Customized ECMO systems are highly adaptable, allowing healthcare professionals to tailor the support for the specific patient’s needs. 

The related mortality rate may be influenced by patients’ clinical presentations at the time of VA-ECMO placement and by the leading cause of CS. 

In their meta-analysis, Alba C. et al. evaluated the different mortality rates following VA-ECMO placement across multiple etiologies of CS. In particular, the mortality rate was 60% (95% CI: 57–64) after AMI. The differences in the mortality rate across the multiple etiologies of CS do not significantly depend on differences in patient’s age and sex [7].

The present narrative review aims to provide an overview of the role of VA-ECMO in CS complicating AMI with the most recent literature highlights. 

## 2. VA-ECMO: Indications, Positioning and Management

VA-ECMO placement is recommended for refractory CS as a lifesaving treatment in several guidelines and consensus documents [8] (Table 1).

It is often considered when conventional treatments, such as intravenous medications and IABP, fail. In cases in which high-risk coronary interventions are planned, VA-ECMO can be initiated as a preemptive measure, providing a safety background therapy in cases of unforeseen complications.

Based on findings from observational studies, current international guidelines and scientific statements endorse a strategy to manage an extracorporeal MCS, although these recommendations are supported by weak levels of evidence [9].

According to the European Heart Failure guidelines, short-term percutaneous MCS are recommended for selected patients with refractory CS (class of recommendation IIa, level of evidence C).

By contrast, American guidelines suggest considering VA-ECMO use more specifically in cases in which a refractory cardiac arrest (CA) occurs [10,11].

The Extracorporeal Life Support Organization (ELSO) suggests developing locally agreed-upon inclusion criteria to assist clinicians in allocating resources to patients deemed to have a higher likelihood of survival after CA. Protocols and guidelines aim to identify cases most likely to achieve favorable neurological outcomes. These include patients with witnessed CA and prompt initiation of high-quality cardiopulmonary resuscitation (CPR), as well as those with CA stemming from potentially reversible causes like acute coronary occlusions.

An example of inclusion criteria for extracorporeal cardiopulmonary resuscitation (ECPR) is reported in Table 1 [12].

Contraindications to VA-ECMO include irreversible organ dysfunction, untreated bleeding disorders, severe neurological injury, advanced age with poor performance status, terminal illness, significant aortic insufficiency or dissection, severe peripheral vascular disease, irreversible pulmonary hypertension, active infections or sepsis, and limited resources. These factors should be carefully considered before initiating VA-ECMO [8].

Cannulation for VA-ECMO typically involves percutaneous or surgical placement of two large-bore cannulas, one for drainage (venous) and one for return (arterial). Common cannulation sites include (1) the femoral vein, which is advanced into the inferior vena cava (IVC), and (2) the femoral or axillary artery, used for arterial return. In some cases, central cannulation involving the right atrium and ascending aorta may be used for direct cardiac support, especially in postcardiotomy V-A ECMO [13].

Hemodynamic monitoring is crucial to make ECMO treatment efficient. It should include (1) tissue perfusion monitoring (mean arterial pressure, lactate level, urine output, liver function); (2) venous drainage monitoring (right heart preload, central venous pressure and pulmonary arterial pressure, WEDGE pressure); (3) systemic and pulmonary arterial pulsatility monitoring; (4) left ventricular (LV) afterload monitoring (systemic vascular resistance); (5) brain perfusion monitoring (avoiding Harlequin Syndrome is recommended); (6) distal leg perfusion monitoring (using a distal perfusion cannula to prevent limb ischemia in peripheral VA-ECMO is recommended); and (7) chest X-ray daily assessment (establishing an LV unloading system should be considered if evidence of pulmonary edema occurs on a chest X-ray or severe mitral insufficiency persists at the echocardiographic assessment) [14].

The complications of VA-ECMO can indeed negatively impact short- and long-term patient outcomes. The most commonly reported complications from the EuroELSO registry include bleeding, thrombosis, limb ischemia, infections, hemolysis, and complications related to cannulation sites. Close monitoring and management of complications are crucial in patients supported with VA-ECMO in order to optimize clinical outcomes and minimize morbidity and mortality [15,16].

Cannulation for VA-ECMO in the setting of AMI should involve a multidisciplinary team including cardiologists, cardiac surgeons, perfusionists, and critical care specialists. Collaboration among team members is essential to optimize patient selection, cannulation techniques, management of complications, and decision-making regarding escalation or de-escalation of mechanical circulatory support.

## 3. New Updates and Highlights about the Role of VA-ECMO in CS

The literature about VA-ECMO in CS complicating AMI was limited to observational retrospective studies until 2019 [17,18,19].

In 2019, Brunner S et al. conducted the first randomized trial involving 42 patients with CS complicating AMI. These patients were randomly assigned to either receive extracorporeal life support (ECLS) or not. Despite expectations, the primary endpoint of the study, i.e., the left ventricular ejection fraction (LVEF) at 30 days, showed similar results between surviving patients in both ECLS (50.0%) and the control group (50.8%). However, the 30-day all-cause mortality was lower in the ECLS group (19%) compared to the control group (33%) [20]. 

The need for more randomized controlled trials on this topic has given rise to the three largest studies analyzing the impact of VA-ECMO on the mortality of patients with CS complicating AMI [21] (Table 2).

### 3.1. EURO-SHOCK Trial

In this trial, patients were randomized to receive VA-ECMO as soon as possible, within 6 h from randomization, or to continue standard therapy. The primary outcome of 30-day all-cause mortality occurred in 43.8% of patients randomized to receive the VA-ECMO and in 61.1% of patients randomized to receive standard therapy (*p* = 0.22). Among the patients in the VA-ECMO group, five of them did not receive VA-ECMO therapy due to complications on the vascular access site or difficulties with peripheral cannulation. Additionally, one patient from the standard therapy group received VA-ECMO due to clinical deterioration. All-cause mortality at 12 months was numerically lower in the VA-ECMO group (51.8% vs. 81.5%: *p* = 0.14). However, the overall recruitment to the trial was significantly impacted by the COVID-19 pandemic, and less than 10% of the initially planned recruitment was completed. As a result, no definitive conclusions can be drawn from these data [22].

### 3.2. ECMO-CS Trial 

The ECMO-CS trial aimed to compare the immediate implementation of VA-ECMO with early conservative therapy, if hemodynamic deterioration occurred. The 30-day all-cause mortality was 50.0% versus 47.5% in the early and delayed VA-ECMO groups, respectively, with a risk difference of 2.5 (95% CI, −15.6 to 20.7). 

A significant portion (39%) of patients in the delayed VA-ECMO group subsequently required VA-ECMO or other forms of MCS due to further hemodynamic deterioration.

However, implementing VA-ECMO immediately in patients with rapid deterioration did not result in improved clinical outcomes. 

In this study, patients were enrolled regardless of the CS origin, even if the most common cause of CS in both arms was ST-segment elevation AMI (50% in both groups). Therefore, the main result of this study is that in patients experiencing severe or rapidly deteriorating CS, managing hemodynamic stabilization with inotropes and vasopressors and reserving MCS for cases of further hemodynamic deterioration produced similar outcomes as in cases when VA-ECMO was positioned immediately [23].

### 3.3. ECLS-SHOCK Trial

This is the largest randomized controlled trial on VA-ECMO in patients with CS complicating AMI, and the aim was to assess whether the addition of early ECLS to early revascularization could have offered better outcomes when compared to early revascularization alone, with the option of transitioning to other MCS devices if required.

Mortality at 30 days was 47.8% in the ECLS group and 49.0%in the control group (relative risk 0.98 (95% CI, 0.80 to 1.19; *p* = 0.81)) [24]. A subgroup analysis revealed potential benefits in patients with non-ischemic CS. 

This study has the highest statistical power considering mortality as a primary endpoint.

According to the Kaplan–Meier curve on all-cause mortality at 30 days, those who received VA-ECMO in the first ten days initially appeared to be better, but then they died at the same rate as those who did not undergo a VA-ECMO placement. 

It is important to highlight the concept that approximately 50% of all-cause mortality at 30 days was due to the events related to the CS itself, in both groups and not to the VA-ECMO. (see ECLS-SHOCK trial Appendix). The mean duration of VA-ECMO running was 2.7 days in all patients undergoing ECLS placement in each group.

On the other side, ECLS was associated with a higher incidence of complications, including moderate or severe bleeding (23.4% vs. 9.6%) and peripheral vascular complications requiring intervention (11.0% vs. 3.8%). In the ECLS-SHOCK trial, a considerable number of patients underwent CPR before randomization (77.7% in the ECLS group and 77.9% in the control group). However, subgroup analysis does not show differences in mortality between post-CPR patients who received ECLS treatment and those who did not. These findings underscore the importance of personalized treatment approaches and further research to refine patient selection criteria and optimize ECMO management protocols in cardiogenic shock.

## 4. The Role of Left Ventricular Unloading Systems during ECLS

Peripheral VA-ECMO can lead to increased LV afterload due to retrograde aortic flow. 

LV unloading is crucial when associated with VA-ECMO. It helps to mitigate LV distension and improve myocardial recovery by reducing myocardial work and pulmonary congestion. 

Preventing pulmonary congestion also allows the medical team to keep the patient awake, self-breathing, and cooperative, thus reducing the risk of infections and critical illness.

LV unloading can be achieved using IABP or percutaneous ventricular assist devices, such as Impella. 

Recent non-randomized studies suggest the potential benefits of using additional unloading devices alongside ECLS, although they also indicate higher rates of complications such as bleeding, hemolysis, and vascular issues.

Schrage B. et al. found that LV unloading was associated with lower 30-day mortality in CS patients treated with VA-ECMO, despite a higher complication rate (hazard ratio: 0.79 [95% CI, 0.63–0.98]; *p* = 0.03). These findings suggest that incorporating an LV unloading device in the VA-ECMO may be beneficial, and they call for further scientific validation [25].

In the ECLS-SHOCK trial, the adoption of at least one LV unloading strategy was noted in only 5.8% of patients in the ECLS group. Among these strategies, the Impella device was the most commonly used (81.8% in the ECLS group and 66.7% in the control group). The LV unloading strategy was protocolized according to the guidelines provided by ELSO [8].

The implementation of LV unloading strategies in very few cases may have affected the final results of the study, in which the placement of VA-ECMO did not actually improve the 30-day outcomes.

Further randomized trials are needed to evaluate whether the LV unloading systems may really improve the clinical outcomes during ECLS running. 

In a recent meta-analysis, conducted on twelve studies and 5063 patients, Merteens MM et al. have demonstrated that the combination therapy of VA-ECMO + IABP is superior to VA-ECMO only in patients with CS due to AMI [26]. They showed decreased in-hospital mortality at 30 days after VA-ECMO + IABP when compared to VA-ECMO alone for patients with CS after AMI (OR 0.36, 95% CI 0.30–0.44, *p* ≤ 0.001). In addition, a combination of VA-ECMO + IABP was associated with a higher rate of weaning (OR 0.29, 95% CI 0.16–0.53, *p* < 0.001) without an increase in vascular complications (OR 0.85, 95% CI 0.35–2.08, *p* = 0.72).

This meta-analysis, although relevant, is analyzing 12 observational retrospective studies, so these findings should be considered as hypothesis-generating only.

### Left Ventricular Unloading with Impella during VA-ECMO: HOW and WHEN to WEAN

The Impella device is a percutaneous ventricular assist device working as a short-term MCS. It is inserted via a catheter into the LV, where it pumps blood directly from the LV into the ascending aorta. The Impella device is often used in patients with acute left heart failure, CS, or during high-risk percutaneous coronary interventions to guarantee adequate systemic perfusion. It can play a significant role when peripheral VA-ECMO therapy is established. As the VA-ECMO can lead to left ventricular distension and dysfunction, incorporating Impella during VA-ECMO weaning helps to reduce LV workload, improve CO, and enhance myocardial recovery in the weaning from VA-ECMO [27]. The association between peripheral VA-ECMO and Impella is called ECPELLA or ECMELLA. The weaning from ECMELLA starts by gradually decreasing VA-ECMO support while simultaneously keeping Impella support. In this way, clinicians can allow for a smoother transition to native heart function. This approach optimizes patient outcomes by promoting myocardial recovery and minimizing the risk of hemodynamic instability during ECMO weaning. 

Furthermore, the combination of VA-ECMO and Impella can potentially reverse the fibrotic and inflammatory pathways occurring with CS. In particular, it can activate cellular mechanisms of cardio-protection and cardiac repair when used as a prolonged support over several weeks (PROPELLA) [28].

After ECMELLA positioning, inotropes should be quickly weaned (1) to maintain full mechanical unloading; (2) not to obscure a true evaluation of myocardial function; and (3) to allow for a- and b-receptor upregulation for enhanced drug responsiveness post-wean [29].

Among inotropes, the relatively new calcium sensitizer levosimendan should be considered in the weaning phase due to (1) the long-lasting inotropic support to the native heart, and (2) the vasodilatory effect with reduction of pulmonary vascular resistances and improvement of right ventricular function. 

While waiting for the two ongoing randomized trials, LEVOECMO and Weanlevo (NCT04158674), the best available scientific evidence on the beneficial effects of levosimendan in facilitating the weaning from VA-ECMO comes from Marabotti et al. They confirm a higher rate of VA-ECMO weaning and a reduced risk of mortality using levosimendan [30].

One of the main issues associated to ECMELLA are the ideal timing and approach to the weaning process. Since there are no standardized temporary MCS weaning and explant guidelines, the approach to the weaning should consist of a multidisciplinary team exercise.

ECLS weaning is based on the evaluation of circulatory stability with ECMO flow to a minimum rate of 1.2–1.5 L/min and confirmation of multiorgan recovery. Usually, VA-ECMO weaning is the first step. Impella support is usually discontinued in a separate weaning step. A precise assessment of the left heart function is only feasible at the minimal flow (P2) [31,32]. The weaning should be tailored to the ongoing patient’s specific needs in order to maintain hemodynamic stability based on CS severity and pathology.

There are several key concepts to observe during the weaning process. More specifically, we should avoid a “one-size-fits-all” approach. Furthermore, we should keep the time of mechanical unloading as short as possible in order to prevent device-related complications but as long as needed in order to obtain a sustained impact on the LV recovery.

Weaning is not a single event. We should assess the patient’s readiness to wean after any single step of the weaning process. Our target should be not necessarily the full restoration of cardiac function but rather an improvement in clinical, hemodynamic, metabolic, and imaging parameters [33]. The ideal target during the weaning process is the real-time monitoring of left ventricular end-diastolic pressure and the total cardiac power output (the sum of outputs generated by the native heart and left ventricular pump). 

In case of coronary disease, the patient should have received complete revascularization and major valvular dysfunctions should have been addressed before weaning.

In case of unrecoverable heart function, the ECMELLA becomes a bridge to a left ventricular assist device (LVAD) placement. LVAD is a safe and effective strategy in the management of INTERMACS 1 patients [34].

## 5. Discussion and Conclusions

To date, the ECLS-SHOCK trial is the largest randomized controlled trial on VA-ECMO in patients with CS complicating AMI. 

However, the outcomes of the ECLS-shock trial and the EURO-SHOCK trial are similar to those published previously. Further studies are needed to fix the ideal timing and indications for VA-ECMO placement in these kinds of patients [22,24].

So, an “early” and “routine” use of VA-ECMO in patients with CS from acute coronary syndrome cannot be supported due to the lack of strong evidence and the related risks of bleeding and vascular complications. Using VA-ECMO for CS is like opening a parachute. Opening it either too early or above a critical altitude after confirming that the individual cannot fly brings to similar outcomes; the individual survives but he may face complications. Conversely, if the parachute is opened too late or not at all, the outcome is predetermined, although few are going to enroll patients in a trial in which they receive no parachute anymore [35].

We are waiting for the results of the two following ongoing randomized trials regarding the management of AMI complicated by CS:

- Anchor trial (NCT04184635) (started in October 2021). The study is designed to examine whether the combination of VA-ECMO support and IABP results in improved clinical outcomes when compared to medical treatment alone in patients with AMI and CS;
- Recover IV trial (NCT05506449) (started in October 2023) The aim of this study is to evaluate whether initiating a hemodynamic support strategy using Impella before percutaneous coronary intervention (PCI) in patients with ST-segment elevation myocardial infarction (STEMI) complicated by CS leads to better survival and functional outcomes when compared to a treatment strategy without Impella.

The Danger Shock trial, which included 355 patients with AMI complicated by CS, has recently demonstrated a notable survival benefit for those treated with the Impella device in addition to standard care. Indeed, a 13% reduction in six-month mortality for patients treated with Impella compared to standard care (45.8% vs. 58.5% mortality) has been demonstrated. Such results mark a significant advancement in the treatment of AMI, providing evidence that the Impella device can improve survival in this high-risk patient population when used appropriately. In particular, subgroup analyses suggested that the survival benefit was more pronounced in patients with very low blood pressure (mean arterial pressure ≤ 63 mmHg) and those with multivessel disease [36].

There is a strong need to standardize the use of the LV unloading systems in CS complicating AMI patients, alone or in addition to the peripheral VA-ECMO. In the papers mentioned above, the use of LV unloading systems is not homogeneous across the study groups, and the indications for their use are not standardized.

A recent trial aimed to assess the impact of ECMELLA compared to VA-ECMO with or without IABP in patients with refractory CS, including cardiac arrest. The ECMELLA group was associated with improvement in short- and long-term mortality [37]. However, this is a single-site cohort study and stronger scientific evidence is needed.

To resume, standardizing the timing and indications for MCS start in CS complicating AMI is crucial and should guide future trials in this field, in order to understand also if the routine additional use of LV unloading systems can make a difference in the patient’s clinical outcomes.

Above these considerations, the approach should always be multidisciplinary and tailored to the specific patient’s needs. The overall purpose should always consist of limiting the complications occurring from unnecessary initiations of MCS devices i.e., “primum non nocere”.

## Figures and Tables

**Table 1:** ELSO criteria for VA-ECMO.

**When to consider VA-ECMO:**
- within 6 h from CS occurrence, if refractory to conventional pharmacological and fluid therapy; - in patients with reversible cardiocirculatory collapse or those eligible for alternative cardiocirculatory assistance, for example, ventricular assist devices (VADs) or transplantation; - etiologies compromising appropriate ECMO function (aortic insufficiency) should be considered to represent potential contraindications; - age, per se, should not be used as an absolute contraindication; - poor life expectancy, severe liver disease, acute brain injury, vascular disease, and immunocompromise represent exclusion criteria for ECMO application.
**Example of inclusion criteria for ECPR:**
- age under 70 years; - witnessed cardiac arrest; - cardiac arrest to first CPR (“no flow time”) < 5 min; - initial cardiac rhythm of VF/pVT/PEA; - arrest to ECMO flow (“low flow time”) < 60 min; - end-tidal CO_2_ > 10 mmHg during CPR; - intermittent ROSC or recurrent VF.

ELSO, European Life Support Organitation; VA-ECMO, veno-arterial extracorporeal membrane oxygenation; CS, cardiogenic shock; ECPR, extracorporeal pulmonary resuscitation; CPR, cardiopulmonary resuscitation; VF, ventricular fibrillation; pVT, pulseless ventricular tachycardia; PEA, pulseless electrical activity.

**Table 2:** Comparative table of studies on the impact of VA-ECMO on survival.

Authors	Year of Randomization	Type of Study	Patient Population	Primary Endpoint	Group	Size	LV unloading Management	30 Day—Mortality
Brunner S. et al. [20]	2019	monocentric, open-label, randomized controlled	CS complicating AMI *	To assess the effect of VA-ECMO on 30-day mortality	ECLS group	21	not reported	19% in the ECLS group and 33% in the control group. (*p* = 0.37)
					no-ECLS group	21		
Banning A.S. et al. (EURO-SHOCK trial) [22]	January 2020—January 2022	multicentric, open-label, randomized controlled	CS complicating AMI **	To assess the effect of VA-ECMO on 30-day mortality	ECLS group	17	IABP in all VA-ECMO patients	43.8% in the ECLS group and 61.1% in the control group. (*p* = 0.22)
					no-ECLS group	18		
Ostadal P. et al. (ECMO-CS trial) [23]	September 2014—January 2022	multicentric, open-label, randomized controlled	all non-surgical causes of CS ***	To compare the immediate implementation of VA-ECMO with early conservative therapy	immediate ECMO	58	LV unloading at the discretion of the physician	50% in the immediate ECMO and 47.5% in the early conservative group.
					early conservative therapy	59		
Thiele H. et al. (ECLS-SHOCK trial) [24]	June 219—November 2022	multicentric, open-label, randomized controlled	CS complicating AMI ****	To assess the early addition of ECLS to early revascularization	ECLS group	211	Unloading rate in ECLS group 5.8%	47.8% in the ECLS group and 49.0% in the control group (*p* = 0.81).
					no-ECLS group	209		

ECLS, extracorporeal life support; CS, cardiogenic shock; AMI, acute myocardial infarction; ECMO, extracorporeal membrane oxygenation; IABP, intra-aortic balloon pump; ROSC, return of spontaneous circulation; SCAI, Society for Cardiovascular Angiography and Interventions; LV, left ventricle. * 95% of patients had been resuscitated before randomization. ** 50% of patients had been resuscitated before randomization. **** ROSC patients were 77.5% in the ECLS group and 77.9% in the no-ECLS group. * and ** CS was considered if systolic blood pressure was less than 90 mmHg for more than 30 min or needed infusion of catecholamines to maintain a systolic pressure above 90 mm Hg, clinical signs of pulmonary congestion, and impaired ex-end organ perfusion. *** and **** CS was considered if SCAI stage D–E.

## Data Availability

Not applicable.
